# Intake of Fruit Juice and Incidence of Type 2 Diabetes: A Systematic Review and Meta-Analysis

**DOI:** 10.1371/journal.pone.0093471

**Published:** 2014-03-28

**Authors:** Bo Xi, Shuangshuang Li, Zhaolu Liu, Huan Tian, Xiuxiu Yin, Pengcheng Huai, Weihong Tang, Donghao Zhou, Lyn M. Steffen

**Affiliations:** 1 Department of Maternal and Child Health, School of Public Health, Shandong University, Jinan, China; 2 Department of Epidemiology and Health Statistics, School of Public Health, Shandong University, Jinan, China; 3 Division of Epidemiology and Community Health, School of Public Health, University of Minnesota, Minneapolis, United States of America; 4 Department of Endocrinology, Linyi People's Hospital, Linyi, China; Geisel School of Medicine at Dartmouth College, United States of America

## Abstract

**Background:**

Several prospective studies have been conducted to examine the relationship between fruit juice intake and risk of incident type 2 diabetes, but results have been mixed. In the present study, we aimed to estimate the association between fruit juice intake and risk of type 2 diabetes.

**Methods:**

PubMed and Embase databases were searched up to December 2013. All prospective cohort studies of fruit juice intake with risk of type 2 diabetes were included. The pooled relative risks (RRs) with 95% confidence intervals (CIs) for highest vs. lowest category of fruit juice intake were estimated using a random-effects model.

**Results:**

A total of four studies (191,686 participants, including 12,375 with type 2 diabetes) investigated the association between sugar-sweetened fruit juice and risk of incident type 2 diabetes, and four studies (137,663 participants and 4,906 cases) investigated the association between 100% fruit juice and risk of incident type 2 diabetes. A higher intake of sugar-sweetened fruit juice was significantly associated with risk of type 2 diabetes (RR = 1.28, 95%CI = 1.04–1.59, *p* = 0.02), while intake of 100% fruit juice was not associated with risk of developing type 2 diabetes (RR = 1.03, 95% CI = 0.91–1.18, *p* = 0.62).

**Conclusions:**

Our findings support dietary recommendations to limit sugar-sweetened beverages, such as fruit juice with added sugar, to prevent the development of type 2 diabetes.

## Introduction

Type 2 diabetes, one of the main causes of morbidity and mortality, has significantly increased worldwide in recent years. Thus, it is important to identify modifiable factors to reduce the risk of developing type 2 diabetes. Although sugar-sweetened beverage (SSB) consumption decreased among youth and adults in the United States between 1999 and 2010 [1], an increased intake of these beverages was observed in Asians [2]. Since the consumption of SSBs has been associated with an increased risk of obesity [3] and type 2 diabetes [4], reduction of SSB intake should be a recommended strategy to promote optimal health. Fruit juice, different from SSBs, has been considered a healthier drink. However, to date, the findings of the association between fruit juice intake and risk of type 2 diabetes are mixed according to type of fruit juice [5]–[11]. Several prospective studies suggested that higher intake of sugar-sweetened fruit juice may increase the risk of developing type 2 diabetes [5], [10], while others showed no significant association for 100% fruit juice [6], [8], [9].

To our knowledge, no meta-analysis has been published to accurately estimate the strength of the effects of type of fruit juice (sugar-sweetened or 100%) on incidence of type 2 diabetes. Although all four studies included in this meta-analysis showed no significant association between 100% fruit juice and risk of developing type 2 diabetes, there was a positive trend for three [6], [8] of the four studies, and insufficient power may explain the non-significant study findings. Therefore, in this study, we conducted a systematic review and meta-analysis to address this issue.

## Materials and Methods

### Literature and search strategy

The PRISMA checklist is available as Checklist S1. The Meta-analysis of Observational Studies in Epidemiology (MOOSE) guidelines were followed for the current study [12]. The literature databases including PubMed and Embase were searched. Search terms were “fruit juice” and “type 2 diabetes” or “T2DM”. The reference lists of retrieved articles were also screened. The literature search was limited to the English language. If more than one article was published on the same cohort, only the study with the largest sample size was included. The literature search was updated on December 10, 2013.

### Inclusion criteria and data extraction

Studies included in the meta-analysis met the following inclusion criteria: (1) evaluation of the association between fruit juice intake and incidence of type 2 diabetes; (2) a prospective study design; and (3) covariate adjusted relative risks (RRs) or hazard ratios (HRs) with 95% confidence intervals (CIs) for highest vs. lowest category of fruit juice intake. The following information was extracted from each study: (1) name of the first author; (2) year of publication; (3) country of study; (4) number of incident cases and study population; (5) age distribution of the study population at baseline; (6) sex of the participants; (7) average duration of follow-up; (8) the covariates included in the regression models; and (9) RRs or HRs with 95% CIs for highest vs. lowest category of fruit juice intake. Two investigators (SL and ZL) independently assessed the articles for compliance with the inclusion/exclusion criteria and resolved disagreements through discussion.

The quality of each study was assessed by the Newcastle–Ottawa quality scale (NOS) [13], which is a validated scale for non-randomized studies in meta-analyses. This scale assigned a maximum of nine points for each study. Three broad perspectives were considered: the selection of the cohorts (4 points); the comparability of cohorts (2 points); and the ascertainment of the exposure and outcome of interest (3 points).

### Statistical analysis

A random effects model [14] was used to calculate pooled RRs with 95% CIs for highest vs. lowest category of fruit juice intake. Heterogeneity was assessed by the Q test and the *I* ^2^ statistic [15]. The significance for the Q test was defined as *p*<0.10. The *I* ^2^ statistic represents the amount of total variation attributed to heterogeneity. Low, moderate, and high degrees of heterogeneity correspond to *I* ^2^ values of 25%, 50%, and 75%, respectively. Publication bias was assessed by Begg's test [16] and Egger's test [17] (*p*<0.05 was considered statistically significant). Statistical analysis was conducted using STATA version 11 (StataCorp LP, College Station, TX, USA).

## Results

### Study characteristics

**Figure 1:**
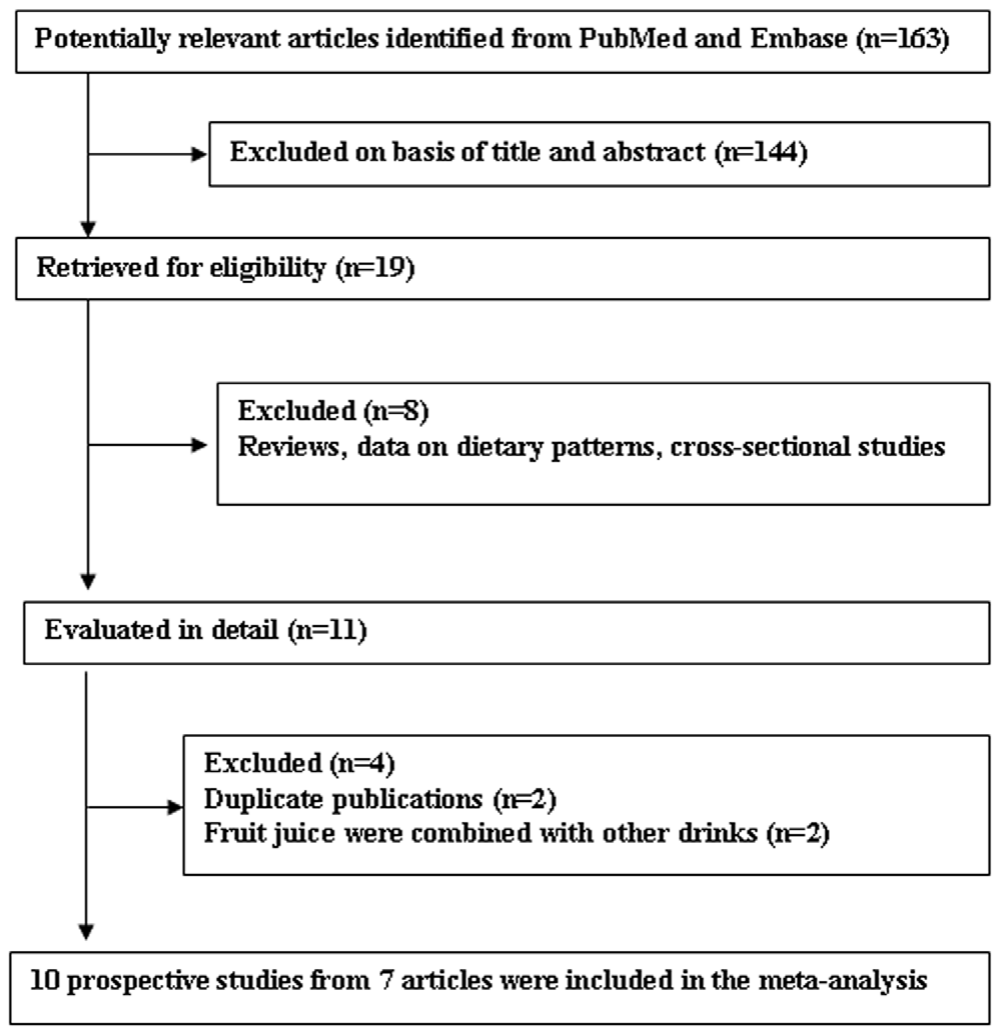
Process of study selection.

Figure 1 shows the process of study selection for the meta-analyses. It should be noted that two duplicate publications [18], [19] and two publications [20], [21] that combined fruit juices with other drinks were excluded. A total of 10 prospective studies from 7 publications were included in the meta-analysis of fruit juice intake and risk of developing type 2 diabetes [5]–[11]. In the publication by Eshak et al. [8], results were reported for each gender and were handled as two independent studies. In addition, the publication by Muraki et al. [10] contained three studies but provided pooled estimate based on them. The characteristics of the studies included in the meta-analyses are summarized in Table 1. Among all studies included in the meta-analysis, four studies examined sugar-sweetened fruit juice [5], [10], four studies examined 100% fruit juice [6], [8], [9], and two studies did not specify the type of fruit juice [7], [11]. Four studies were from the USA [6], [10], three from Europe [5], [9], and three from Asia [7], [8]; and the duration of follow-up ranged from 5.7 to 25 years.

**Table 1:** Characteristics of included studies on fruit juice intake and risk of type 2 diabetes.

Study	Country	No. of cases/ participants	Sex	Age (years)	Follow-up (years)	Dietary assessment (juice type)	Diagnosis of type 2 diabetes	RR (95%CI) for highest vs. lowest intakes	Confounders' Adjustment	Study quality^a^
Montonen et al, 2007 [5]	Finland	177/4304	Men and women	40–60	12	Dietary history interview (sugar-sweetened)	Confirmed via social insurance institutions register	51 g/day vs. 0 g/day: 1.56 (1.08–2.26)	Age, sex, BMI, energy intake, smoking, geographic area, physical activity, family history of diabetes, prudent dietary pattern score, conservative pattern score, serum cholesterol, blood pressure, history of infarction, history of angina pectoris, and history of cardiac failure	7
Palmer et al, 2008 [6]	USA	2713/43960	Women	38±10	10	FFQ (100%)	Self-reported and validated in a random sample	≥2 drinks/day vs. <1 drink/month: 1.11 (0.92–1.35)	Age, family history of diabetes, physical activity, cigarette smoking, years of education, each of the 2 other types of drinks, intake of red meat, processed meats, cereal fiber, and coffee, and glycemic index	7
Odegaard et al, 2010 [7]	Singapore	2273/43580	Women	45–74	5.7	FFQ (unknown)	Confirmed via hospital databases	2–≥3 glasses/week vs. never: 1.24 (1.01– 1.53)	Age, sex, dialect, year of interview educational level, smoking status, alcohol use, physical activity, saturated fat intake, dietary fiber intake, dairy intake, juice or soft drink intake depending on model, coffee consumption,BMI, energy intake, weight gain	7
Eshak et al, 2013 [8]	Japan	824/27585	Men and women	40–59	10	FFQ (100%)	Self-reported and validated in a random sample	Men: 1 time/day vs. rarely: 1.17 (0.69–2.00) Women: 1 time/day vs. rarely: 1.37 (0.79–2.37)	Age, BMI, family history of diabetes mellitus, education, occupation, smoking status, alcohol intake, history of hypertension, leisure-time physical activity, consumption of coffee, consumption of green tea, energy-adjusted intakes of dietary magnesium, calcium, vitamin D, rice and total dietary fiber, and total energy intake	7
Fagherazzi et al, 2013 [9]	France	1369/66118	Women	53±7	14	FFQ (100%)	Confirmed via health insurance records and questionnaire	>967 ml/week vs. none: 0.93 (0.78–1.10)	Years of education, smoking status, physical activity; hypertension, hypercholesterolemia, use of hormone replacement therapy, family history of diabetes, self-reported use of antidiabetic drugs, alcohol intake,omega-3 fatty acid intake, carbohydrate intake, coffee, fruit and vegetables, processed-meat consumption, dietary pattern, total energy intake and BMI	7
Muraki et al, 2013 [10]	USA	NHS: 6358/66105 NHS II: 3153/85104 HPFS: 2687/36173	Men and women	NHS: 52–77 NHS II: 35–52 HPFS: 40–75	NHS: 25 NHS II: 9 HPFS: 23	FFQ (sugar-sweetened)	Self-reported and validated in a random sample	≥1 serving/day <1 serving/week: 1.21 (1.13–1.29)	Age, ethnicity, BMI, smoking status, multivitamin use, physical activity, family history of diabetes, menopausal status and post-menopausal hormone use, oral contraceptive use (for Nurses' Health Study II), total energy intake, fruit juice consumption and the modified alternate healthy eating index score	8
Mursu et al. 2013 [11]	Finland	432/2332	Men	42–60	19.3	Instructed 4–d food recording (unknown)	The National hospital discharge registry and Social Insurance Institution of Finland reimbursement registry	Q4 vs. Q1: 0.99 (0.74–1.31)	Age, examination years, BMI,waist-to-hip ratio, smoking, education, leisuretime physical activity, family history of diabetes, and intakes of energy and alcohol	7

NHS, Nurses' Health Study; HPFS, Health Professionals Follow-up Study; FFQ, food-frequency questionnaire; BMI, body mass index.

a The quality of each study was assessed by Newcastle–Ottawa quality scale.

### Meta-analysis of fruit juice intake and risk of developing type 2 diabetes

**Figure 2:**
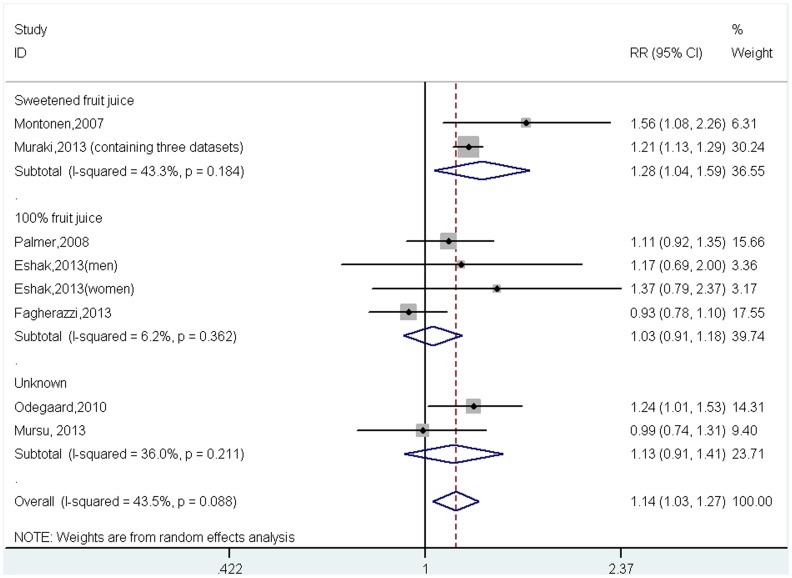
Relative risk for incident type 2 diabetes for highest versus lowest intake of fruit juice (by type of juice).

A total of 375,261 participants, including 19,986 with incident type 2 diabetes, were included in the meta-analysis. The pooled results indicate that individuals with a higher intake of fruit juice had a greater risk of developing type 2 diabetes (RR = 1.14, 95%CI = 1.03–1.27 *p* = 0.01, Figure 2), with modest evidence of between-study heterogeneity (*I* ^2^ = 43.5%, *p* = 0.09).

To determine if the risk of developing type 2 diabetes was different for fruit juice with added sugar than for 100% fruit juice, subgroup analyses were conducted stratified for sugar-sweetened fruit juice and 100% fruit juice. Four studies (191,686 participants, including 12,375 with type 2 diabetes) investigated the association between sugar-sweetened fruit juice and risk of developing type 2 diabetes, and four studies (137,663 participants and 4,906 cases) investigated the association between 100% fruit juice and risk of type 2 diabetes. Notably, higher intake of sugar-sweetened fruit juice was significantly associated with greater risk of incident type 2 diabetes (RR = 1.28, 95%CI = 1.04–1.59, *p* = 0.02, *I* ^2^ = 43.3%, *p* = 0.184, Figure 2), while there was no association between intake of 100% fruit juice and risk of incident type 2 diabetes (RR = 1.03, 95%CI = 0.91–1.18, *p* = 0.62, *I* ^2^ = 6.2%, *p* = 0.362, Figure 2).

Since the publication by Muraki et al. [10] did not provide the study-specific result for each of the three studies included in their meta-analysis, we were unable to assess the effects of the other characteristics of the participants in these studies, such as age, gender, origin of country, follow-up time, whether or not BMI was adjusted for the association between sugar-sweetened fruit juice and type 2 diabetes. But we did assess those variables for the other four studies for the association between 100% fruit juice and incident type 2 diabetes. As the age of the study population and follow-up time were similar between these four studies, we only assessed the effects of gender, origin of country, and whether or not BMI was adjusted. The results remained non-significant for 100% fruit juice in both sexes (men: RR = 1.17, 95%CI = 0.69–1.99; women: RR = 1.02, 95%CI = 0.90–1.16), in each country (USA: RR = 1.11, 95%CI = 0.92–1.34; Japan: RR = 1.26, 95%CI = 0.86–1.85; France: RR = 0.93, 95%CI = 0.78–1.10), and in both models with or without adjustment for BMI (without adjustment for BMI: RR = 1.11, 95%CI = 0.92–1.34; adjusted for BMI: RR = 0.98, 95%CI = 0.84–1.15).

### Potential publication bias

**Figure 3:**
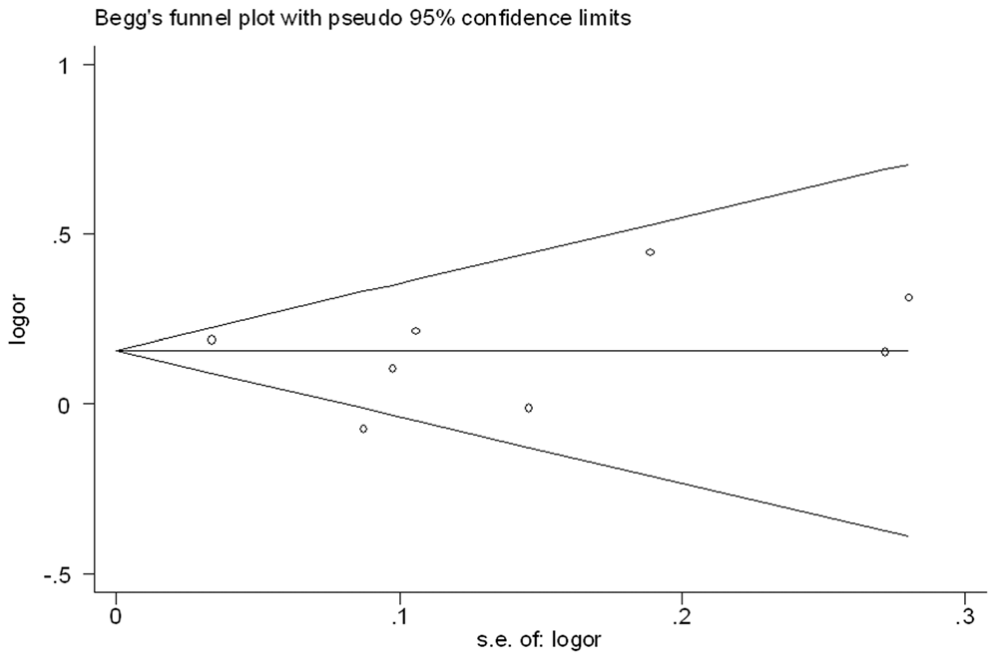
Funnel plot of the association between intake of fruit juice and incident type 2 diabetes.

No publication bias was detected (*p* = 0.71 for Begg's test and *p* = 0.77 for Egger's test; Figure 3).

## Discussion

In the present study, we first performed a systematic meta-analysis to investigate the association between fruit juice intake and risk of incident type 2 diabetes. Results from our meta-analysis suggested that a greater intake of fruit juice was associated with a 14% higher risk of incident type 2 diabetes. However, in subgroup analyses, only sugar-sweetened fruit juice intake was associated with an increased risk of developing type 2 diabetes while 100% fruit juice had no effect.

Two previous meta-analyses showed no association between higher intake of whole fruit and incident type 2 diabetes [22], [23]. However, one recent publication including three large-scale prospective studies showed that a higher intake of whole fruit decreased risk of type 2 diabetes (≥3 servings/day vs.<4 servings/week: RR = 0.88, 95%CI = 0.81–0.96) [10]. An updated meta-analysis is warranted to clarify these inconsistent results.

In the Nurses' Health Study II, fruit punch, a sugar-sweetened fruit drink but different from fruit juice, was associated with a greater risk of incident type 2 diabetes [18]. Indeed, higher consumption of SSBs, including soft drinks, fruit drinks, iced tea, and energy and vitamin water drinks, was associated with development of type 2 diabetes in a previous meta-analysis [4]. Just recently, the relation between fruit juice intake and type 2 diabetes has received more attention. Based on our findings, sugar-sweetened fruit juice had a similar deleterious metabolic action as SSBs in the development of type 2 diabetes. First, the beneficial components of whole fruit, such as naturally occurring soluble fiber, vitamins, minerals and phytochemicals, might have been destroyed or diminished in processing. Second, the high glycemic load of added sugars in beverages may increase the risk of developing type 2 diabetes [24]. In addition, liquid calories may result in more rapid and larger changes in serum levels of glucose and insulin than whole fruit [10].

A previous systematic review reported no association between 100% fruit juice intake and risk of obesity in children and adolescents [25]. This finding is consistent with ours that 100% fruit juice had no effect on the risk of developing type 2 diabetes. The mechanism underlying this association is unclear but two points might explain the finding. First, for sugar-sweetened fruit juice, the healthy components of whole fruit may be destroyed during the processing stage; second, the naturally occurring sugars in 100% fruit juice may have different metabolic effects than added sugars [6], [18].

Our study has several strengths, including the prospective study design, large sample size, long follow-up duration, and relatively precise RRs (95%CIs) adjusted for potential confounders in the studies included in the current meta-analysis. However, several limitations should be considered. First, only several publications were included in the present meta-analysis. However, the total sample size for both sugar-sweetened fruit juice and 100% fruit juice subgroups was relatively large. Second, although most known confounding factors have been adjusted for, we cannot rule out the effect of residual confounding on the observed association. Third, all studies in the meta-analysis used fruit juice intake assessed at baseline; however, it is possible that individuals may have changed their intake of fruit juice during the follow-up period. However, the non-differential misclassification tends to attenuate the observed association towards the null. Fourth, we were unable to determine a dose-response association between fruit juice intake and incidence of type 2 diabetes since different units, e.g., kilogram, milliliter, glass, drink or time, were used to quantify the amount of juice intake. In addition, although we performed the meta-analysis of high intake of fruit juice compared with low intake, the quantities of high and low intakes among the studies may be different. Finally, potential publication bias might influence our results even though Begg's and Egger's tests were not significant.

In conclusion, the present meta-analysis showed that 100% fruit juice intake was not associated with the risk of developing type 2 diabetes; but, a higher intake of sugar-sweetened fruit juice was associated with an increased the risk of incident type 2 diabetes. Our findings have important public health implications. Sugar-sweetened fruit juice is not a healthy choice to replace SSBs, and individuals should limit their intake of sugar-sweetened fruit juice [26] to prevent the development of type 2 diabetes.

## Supporting Information

Checklist S1 Supporting PRISMA checklist.
(DOC)
